# Shifting in the Dominant Bacterial Group *Endozoicomonas* Is Independent of the Dissociation With Coral Symbiont Algae

**DOI:** 10.3389/fmicb.2020.01791

**Published:** 2020-07-31

**Authors:** Jia-Ho Shiu, Sheng-Ping Yu, Chia-Ling Fong, Jiun-Yan Ding, Chih-Jui Tan, Tung-Yung Fan, Chih-Ying Lu, Sen-Lin Tang

**Affiliations:** ^1^Molecular and Biological Agricultural Sciences Program, Taiwan International Graduate Program, Academia Sinica, Taipei, Taiwan, and National Chung-Hsing University, Taichung, Taiwan; ^2^Biodiversity Research Center, Academia Sinica, Taipei, Taiwan; ^3^Graduate Institute of Biotechnology, National Chung-Hsing University, Taichung, Taiwan; ^4^National Museum of Marine Biology and Aquarium, Pingtung, Taiwan; ^5^Department of Oceanography, National Sun Yat-sen University, Kaohsiung, Taiwan

**Keywords:** coral microbe, coral bleaching, *Endozoicomonas*, Symbiodiniaceae, dark treatment

## Abstract

The coral-associated *Endozoicomonas* are dominant bacteria in the coral holobiont. Their relative abundance usually decreases with heat-induced coral bleaching and is proposed to be positively correlated with Symbiodiniaceae abundance. It remains unclear whether this phenomenon of decreased *Endozoicomonas* abundance is caused by temperature stress or a decreased abundance of Symbiodiniaceae. This study induced bleaching in the coral *Euphyllia glabrescens* using a dark treatment over 15 weeks. We examined shifts in *Endozoicomonas* abundance and experimentally reduced Symbiodiniaceae density. 16S rRNA gene amplicon sequencing was used to characterize the changes in bacterial community (incl. *Endozoicomonas*) over time, and the 16S rRNA gene copy number of *Endozoicomonas* was quantified by qPCR. We detected a high abundance of *Endozoicomonas* in *E. glabrescens* that underwent dark-induced bleaching. The results reveal that changes in the relative abundance of *Endozoicomonas* are unrelated to Symbiodiniaceae abundance, indicating that *Endozoicomonas* can be independent of Symbiodiniaceae in the coral holobiont.

## Introduction

Coral-associated bacteria are commonly understood to affect coral health (Bourne and Webster, 2013; Bourne et al., 2016). The diversity and community structure of these bacteria change during coral bleaching, which occurs when corals experience stress that results in the decrease in Symbiodiniaceae abundance (Bourne et al., 2008). The bacteria genus *Endozoicomonas*, a putative core coral bacterial group (Bourne et al., 2016; Neave et al., 2016), is highly associated with coral bleaching. Genome analyses have revealed *Endozoicomonas* to function in DMSP degradation, testosterone degradation, probiotic mechanism, and the Embden-Meyerhof-Parnas (EMP) glycolytic pathway (Neave et al., 2014; Ding et al., 2016; Tandon et al., 2020). In addition, they can constitute >90% of the total bacterial community abundance (Pogoreutz et al., 2018; Shiu et al., 2018). Several studies have described a decrease in *Endozoicomonas* populations in corals during bleaching (Lee et al., 2015; Pantos et al., 2015; Glasl et al., 2016; Ziegler et al., 2017) and a subsequent increase in abundance after corals recover (Bourne et al., 2008). Therefore, *Endozoicomonas* abundance is hypothesized to be positively correlated with Symbiodiniaceae density (Bayer et al., 2013; Neave et al., 2017).

However, in previous studies, Symbiodiniaceae decreased as the result of stress from increased seawater temperature. Hence it is unclear whether the phenomenon of decreased *Endozoicomonas* abundance is caused by temperature stress-associated conditions and/or a decreased abundance of Symbiodiniaceae. In addition, under excessive nutrient treatment, Pogoreutz et al. (2017, 2018) reported that *Endozoicomonas* density remained the same after Symbiodiniaceae abundance decreased, indicating that there was no positive correlation between bacteria and Symbiodiniaceae abundances. Despite this, nutrient levels could have been responsible for the high abundance of *Endozoicomonas* after bleaching. Therefore, taking these inconsistencies into consideration, we tested the hypothesis that *Endozoicomonas* abundance is positively correlated with Symbiodiniaceae density.

We established a non-heat method (Yonge and Nicholls, 1931; William et al., 1982; Hoegh-Guldberg and Smith, 1989; Denis et al., 2012) under oligotrophic conditions to induce bleaching in the coral *Euphyllia glabrescens*. We examined changes in the abundance of *Endozoicomonas* as Symbiodiniaceae decreased during bleaching, induced by a dark treatment over 15 weeks. Throughout the experiment, the abundance of *Endozoicomonas*, total coral-associated bacteria, and Symbiodiniaceae were measured using qPCR. The variation in bacterial community composition was analyzed from 16S rRNA gene amplicon sequencing. Our results contradict the hypothesis, and demonstrate that the abundance of *Endozoicomonas* is not dependent on Symbiodiniaceae density.

## Materials and Methods

### Sample Collection

We collected three colonies of *E. glabrescens* more than 10 m apart at a depth of 3.5 m from the power plant inlet in Kenting, Taiwan (21°57′21.6”N 120°45′18.1″E). *E. glabrescens* is a model species for analyzing different pathways of coral bleaching because the lipogenesis for coral-Symbiodiniaceae endosymbiosis has been studied previously (Chen et al., 2015, 2017). Colonies were transported to a laboratory in Academia Sinica, Taipei within 5 h of collection. One liter of seawater from the collection site was sampled and filtered through a 0.22 μm membrane, then stored at –20°C.

### Bleaching Under the Dark Treatment

**FIGURE 1:**
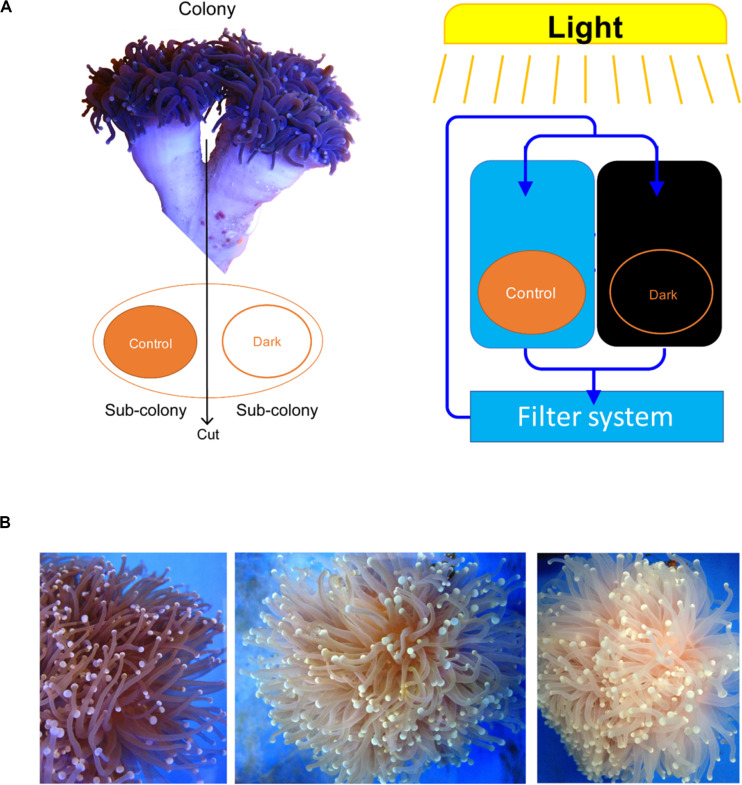
*Euphyllia glabrescence* bleached under the dark treatment. **(A)** Illustration of samples used in the experiment and their arrangement. **(B)** *E. glabrescence* gradually bleached under the dark treatment at week 0, 9, and 12 (from left to right).

Each colony was separated into two fragments, which were placed into two tanks using the same seawater circulation system. After acclimating (1 week), one of the fragments was treated under a light/dark cycle (8 h light/16 h dark) and the other was covered for a dark treatment (24 h dark) for 15 weeks (Figure 1A). There were three seawater circulation systems for three biological repeats. One fragment under the light/dark cycle was not sampled because it was too small to collect its tentacles.

Coral tissue and seawater were serially sampled at week 0, 1, 3, 5, 7, 9, 12, and 15 (Supplementary Table S1). At each sampling week, three coral tentacles from each fragment were cut using scissors and stored in 99% ethanol at −80°C for DNA extraction. A 1-L seawater sample from every circulation system was filtered through a 0.22 μm membrane and stored at −20°C for DNA extraction. Circulation systems were maintained at 23–24°C and monitored using temperature loggers (HOBO© Pendant, Onset Corp, United States) every 15 min throughout the experimental period.

### Total DNA Extraction and Comparative Quantitative PCR (qPCR)

The tentacles stored in 99% ethanol were washed with TE buffer (10 mM Tris-HCl, 1 mM EDTA, pH 8) and ground using a plastic pestle on ice. The homogenized tentacles and seawater-filtered membranes were individually transferred into TE buffer for total DNA extraction using a modified CTAB method (Wilson, 2001; Hong et al., 2009).

Comparative qPCR was used to track changes in the abundance of total bacteria, the *Endozoicomonas* community, and Symbiodiniaceae (Clade C) in coral cells. Four pairs of primers were used to quantify major *Endozoicomonas* populations, total bacterial community, Symbiodiniaceae density in coral, and *Euphyllia*-specific β-actin as an internal control (Table 1). To quantify the variation in Symbiodiniaceae abundance in coral cells, the ITS gene of Clade C Symbiodiniaceae was normalized using the β-actin gene. To quantify the change in *Endozoicomonas* abundance in coral cells, the 16S rRNA gene of *Endozoicomonas* was measured using *Endozoicomonas*-specific primers and normalized using the β-actin gene. To quantify the variation in total bacterial abundance in coral, the 16S rRNA gene in bacteria was quantified using the bacterial universal primers and normalized with the β-actin gene. To determine the dynamics in relative abundance of *Endozoicomonas* to the total bacterial community, the *Endozoicomonas* 16S rRNA gene was measured using *Endozoicomonas*-specific primers and normalized by the 16S rRNA gene of total bacteria.

**TABLE 1:** Characteristics of the primer sets used for qPCR.

Primer	Target	Sequence (5′-3′)	Amplicon (bp)	Annealing temp. (°C)	Efficiency (%)	Range (Ct)	References
En 667F	*Endozoicomonas* 16S rRNA	CTAGAGTGCGGAAGAGGAGT	104^a^	60	99.28	16.53–34.87	this study
mEn771R	*Endozoicomonas* 16S rRNA	TCAGTGTCARRCCAGAGTGT					this study
V6f (967F)	Bacterial 16S rRNA	CAACGCGAAGAACCTTACC	79^a^	60	101.43	12.69–30.59	Chen et al., 2011
V6R (1046R)	Bacterial 16S rRNA	CGACAGCCATGCANCACCT					Chen et al., 2011
SymITS1-QF	Symbiodiniaceae ITS region	TGCGGAAGGATCATTCGCAC	95^b^	60	101.40	12.86–30.45	Chen et al., 2011
SymITSC-QR	Symbiodiniaceae ITS region	CCTCGAGTTCTGCCAGCAGAT					Chen et al., 2011
beta-actin qF	*Euphyllia* beta actin	CGCCTTCCTTGGAATGGAATCCTCT	151^b^	60	100.72	20.72–28.20	Shikina et al., 2016
beta-actin qR	*Euphyllia* beta actin	CTGCATCCTGTCAGCGATTCCAGGG					Shikina et al., 2016

*Characteristics of the primer sets used to identify Endozoicomonas, bacteria, Symbiodiniaceae associated with Euphyllia, and beta-actin of Euphyllia using a qPCR assay on the genomic DNA of Endozoicomonas montiporae^a^ or total coral holobiont DNA extract of sample a^b^ in this study.

**FIGURE 2:**
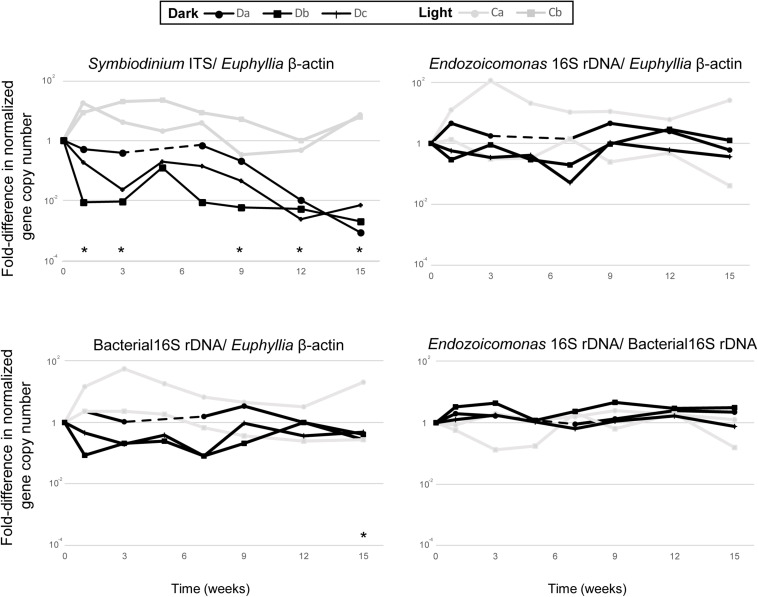
The comparative qPCR results for variation in the abundance of Symbiodiniaceae ITS, *Endozoicomonas* 16S rRNA gene, and total bacteria 16S rRNA gene copy numbers—normalized by *Euphyllia* beta-actin gene copy numbers—and the relative abundance of *Endozoicomonas* 16S rRNA genes normalized by the copy numbers of total bacteria 16S rRNA genes. The black line represents dark-treated samples, and the gray line represents the light-treated samples. Asterisk denotes significant variation (*p* < 0.05) between samples under the dark treatment and samples before treatment. The dashed lines show missing data from the dark treatment at week five.

Each qPCR reaction was performed in 20 μL volumes using the Platinum SYBR Green qPCR SuperMix-UDG Kit (Invitrogen) in a QuantStudio^TM^ five Real-time PCR machine (Applied Biosystems), then analyzed using QuantStudio Design & Analysis software. All qPCR reactions were run using three technical replicates, consisting of 1× platinum SYBR Green qPCR SuperMix UDG, 0.2 μM of each primer, 500 mM ROX reference dye, and 40 ng total DNA template. The qPCR cycling parameters were: initial activation steps at 50°C for 120 s and 95°C for 120 s, followed by 40 cycles of a two-step reaction involving denaturation at 95°C for 15 s and an annealing/extension step at 60°C for 30 s. To confirm that each primer pair produced only one specific product, a melting curve was added to the end of each qPCR assay for each run. Differences in Symbiodiniaceae, *Endozoicomonas* and total bacterial abundance in dark-treated samples were compared with those in the samples before the dark treatment (week 0) by paired *t*-test (Figure 2; *n* = 3).

### Amplification of the Bacterial 16S rRNA Gene and PCR Tagging

The 16S rRNA gene was amplified by PCR with a pair of bacterial universal primers—968F (5′-AACGCGAAGAACCTTAC-3′) and Uni1391R (5′-ACGGGCGGTGWGTRC-3′)—for the bacterial V6–V8 hypervariable region (Nubel et al., 1996; Jorgensen et al., 2012). PCR was performed in 25 μL reaction volumes consisting of 0.75 units TaKaRaEx Taq (Takara Bio, Otsu, Japan), 2.5 μL 1× TaKaRa Ex Taq buffer, 0.2 mM deoxynucleotide triphosphate mixture (dNTP), 0.2 mM of each primer, and DNA samples from coral or seawater. The thermocycler was set to an initial step at 94°C for 3 min; 30 cycles at 94°C for 30 s, 54.3°C for 20 s, and 72°C for 30 s; and a final extension at 72°C for 5 min. PCR amplification was performed three times for each sample, then combined for subsequent analysis. Target DNA bands (∼420 bp) were examined on 1.5% agarose gel after electrophoresis. The amplified samples were eluted using a QIAEX II Gel Extraction Kit (Qiagen, Valencia, CA, United States).

To tag each bacterial V6–V8 amplicon with a unique barcode sequence, each tag primer was designed with four overhanging nucleotides at the 5′ ends of the common primers. The tagging reaction was performed with a 5-cycle PCR, and each cycle was run at 94°C for 30 s, 56°C for 20 s, and 72°C for 45 s with the modified primers. End products were purified by the same gel elution method described above and DNA concentration was determined with a Qubit dsDNA HS assay (Invitrogen, Carlsbad, CA, United States).

### Illumina MiSeq Paired-End Sequencing and Data Processing

We pooled all coral and seawater V6–V8 amplicons equally into two independent libraries and sequenced 2 × 250 paired-end reads with Illumina MiSeq (Yourgene Bioscience, Taipei, Taiwan). High-quality reads were produced after raw reads were merged, sorted, and trimmed using MOTHUR (Schloss et al., 2009) based on the following criteria: (1) reads 350 to 500 bp long, (2) average quality score >27, (3) homopolymer length <8 bp, and (4) reads with any ambiguous base (N) removed. Then the four base tags and primer sequences were removed.

For operational taxonomic unit (OTU) analysis, quality-filtered reads were pooled together and OTUs were assigned at 97% identity with the UPARSE pipeline (Edgar, 2013). Each OTU was classified with a bootstrap value set to 0.8 using a classifier (Wang et al., 2007) against the SILVA database (release 128) implemented in MOTHUR. In UPARSE, de-replication was performed and singletons were excluded (options: –derep_prefix and –minsize 2). Relative abundances of each classified bacterial OTU were calculated for individual samples (Chen et al., 2011). Non-bacterial OTUs were removed in subsequent analyses.

### Data Analyses

To determine the differences in bacterial community compositions between coral and seawater samples, differences in bacterial community composition between coral and seawater samples were tested by global ANOSIM analysis. OTUs assigned to the same order were combined and presented in a bar chart.

To analyze beta diversity and determine the relationships in bacterial OTU communities among samples, the relative abundances of OTUs in individual samples were incorporated into a distance matrix (Bray-Curtis distance). Differences in bacterial communities in coral samples between the two treatments within various sampling times were tested using two-way nested ANOSIM analysis and vice versa, whereas differences among bacterial communities in seawater samples at different sampling times were determined using global ANOSIM.

For variations in *Endozoicomonas* abundance, the average and standard error in the relative abundance of *Endozoicomonas* sequences in coral samples under the dark treatment at each sampling time were calculated and presented in an XY plot. For the most dominant *Endozoicomonas* OTU in each sample, a bubble plot was made using Microsoft^®^ Excel 2016 to determine the relative abundance of each *Endozoicomonas* OTU—except OTU470, which had too low an abundance to be visualized on the plot.

A 16S rRNA gene tree was constructed to estimate the phylogenetic relationships among four representative *Endozoicomonas* OTU sequences and other *Endozoicomonas* reference sequences downloaded from GenBank. The phylogenetic tree was generated using the maximum-likelihood method, with the Tamura-Nei model and 1000 bootstrap replicates, in MEGA7 (Kumar et al., 2016). All base positions containing gaps or missing data in the sequence alignment were discarded. Finally, 405 informative sites in the 23 aligned sequences were available for analysis in the phylogenetic tree.

After excluding the four *Endozoicomonas* OTUs used in the above phylogeny, the 10 most abundant OTUs were selected for heatmap analysis and hierarchical clustering analysis using the default setting—Pearson correlation—in the R package pheatmap (Kolde, 2015).

## Results

### Density of Microbes in Coral Cells During Bleaching

All colonies under dark treatment gradually bleached (Figure 1B and Supplementary Figure S1). Dark-treated samples showed a significant decrease in Symbiodiniaceae abundance over time (paired *t*-test, ex. week 0 vs 15, *p <* 0.001, *n* = 3), and was lower than 1 % by week 12 (Figure 2). No significant variation was detected in the abundance of *Endozoicomonas* over the 15-week dark treatment (ex. week 0 vs 15, *p* = 0.42, *n* = 3; Figure 2), whereas the abundance of total bacteria significantly decreased at week 15 (week 0 vs 15, *p* = 0.01, *n* = 3; Figure 2). No significant variation was detected in the relative abundance of *Endozoicomonas* in total bacteria abundance (ex. week 0 vs 15, *p* = 0.27, *n* = 3; Figure 2).

### Relative Abundance of *Endozoicomonas* in the Total Bacteria Community

**FIGURE 3:**
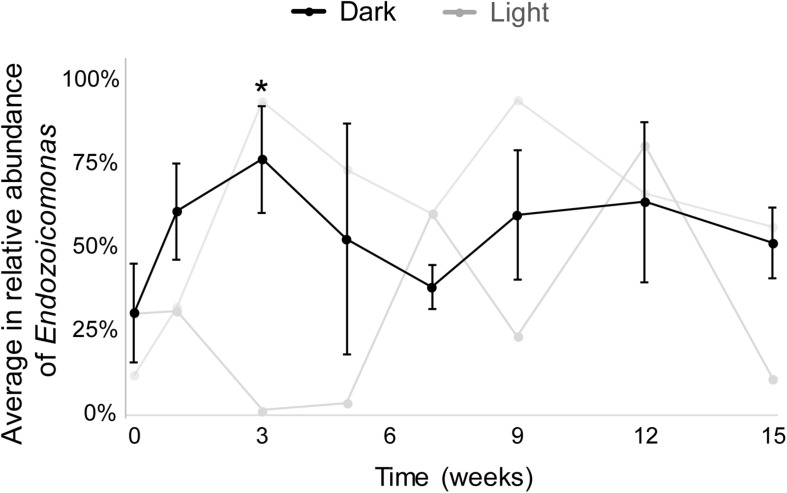
The average relative abundances of *Endozoicomonas* throughout the experimental period. Black lines indicate dark-treated samples and gray lines indicate the system control samples. Standard deviations are marked only in dark-treated samples. Asterisk marks significant variation (*p* < 0.05) between samples under the dark treatment for three weeks and samples before the treatment.

The results from both MiSeq and qPCR datasets were similar (Figure 3). In the MiSeq data, all *Endozoicomonas* OTUs (97% nucleotide sequence identity)—OTU4, OTU9, OTU120, and OTU470 (Supplementary Figures S3A,B)—were combined, and the relative abundance of *Endozoicomonas* was shown after normalization (Figure 3 and Supplementary Figure S2). After 15 weeks of dark treatment, colonies still had high *Endozoicomonas* abundance (relative abundance = 58.5, 55.3, and 38.7% in colony a, b, and c, respectively). There was no significant variation in the relative abundances of samples before and after the dark treatment at each sampling time, except for a significant increase at week 3 (*p* < 0.05; Figure 3). In the comparative qPCR results (Supplementary Figure S2B), there was a similar dynamic in the changes in *Endozoicomonas* 16S rRNA gene copy numbers normalized by the copy number of total bacterial 16S rRNA genes. Both methods found that the *Endozoicomonas* abundance was maintained in the dark-treated colonies after 3 months.

### The Most Abundant *Endozoicomonas* OTUs in Coral Under Darkness

To determine whether any shifting occurred among dominant *Endozoicomonas* OTUs, the relative abundances of OTU4, OTU9, and OTU120 in colonies a, b, and c were shown in a bubble plot (Supplementary Figure S3A). OTU470 was not shown because its relative abundance was <0.005% in all coral samples and therefore too small to show in the bubble plot.

In the light cycle samples, OTU4 was always the most dominant *Endozoicomonas* OTU after week 1, and there was no clear OTU substitution. Under the dark treatment, OTU9 had a higher relative abundance than OTU4 at several sampling times, i.e., weeks 1, 9, 12, and 15 in colony a (Da in Supplementary Figure S3A), and weeks 7 and 9 in colony b (Db). The most abundant OTUs shifted continuously in the a and b colonies, but not in colony c (Dc).

For the phylogenetic tree of the four *Endozoicomonas* OTUs (Supplementary Figures S3B,C), OTU4 had 99.7% identity (407/408 bases) with *E. montiporae* strain CL-33, and OTU9 had 97.7% identity (399/408 bases) with *E. euniceicola* strain EF212. OTU120 had 97.0% identity (396/408 bases) with *Endozoicomonas sp.* strain JOB-63a. OTU470 had 99.5% identity (406/408 bases) with *Endozoicomonas sp.* strain Acr-14.

### Variation in Other Bacterial Communities Under Darkness

Overall, bacterial communities differed between coral and seawater samples (ANOSIM, *R* = 0.907, *p* < 0.001; Supplementary Figure S4A). Regarding order-level variation in the bacterial composition between all coral and seawater samples, *Altermonadales* and *Rhodobacterales* were dominant (29.2 and 27.0% of the relative abundance, respectively) in seawater samples, whereas *Oceanospirillales* was dominant in coral samples (59.3%; Supplementary Figure S4B). In addition, 98.3% of *Oceanospirillales* sequences in coral samples belonged to *Endozoicomonas*.

There was significant variation in the community composition of coral samples between different treatments (2-way nested ANOSIM, *R* = 0.749, *p* < 0.001). To understand the variations in coral-associated bacterial communities other than *Endozoicomonas* during coral bleaching by the dark treatment, bacterial communities from MiSeq data were analyzed after removing all *Endozoicomonas* OTUs.

**FIGURE 4:**
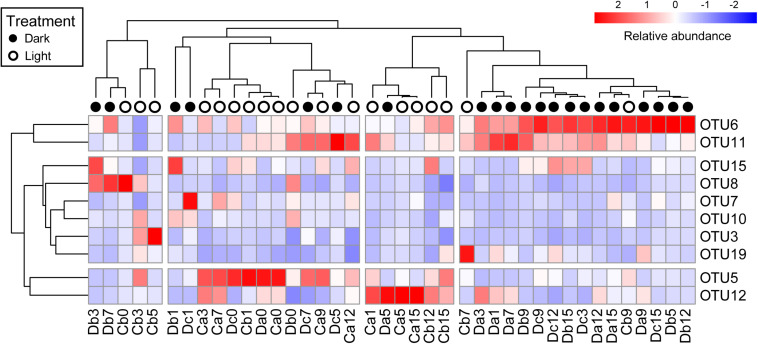
Heatmap of top 10 OTUs in all coral samples after removal of *Endozoicomonas* OTUs. The top 10 OTUs on the *y*-axis comprised >1% relative abundance in all coral samples. Coral samples on the *x*-axis and OTUs on the *y*-axis were clustered based on Pearson correlation. Relative abundance of the top 10 OTUs in samples is presented (after transformation with a *z*-score). Red boxes present higher abundance, whereas blue boxes indicate lower abundance. Sample names on the *x*-axis with black or white circles indicate that the samples were under darkness or light, respectively, regardless of the sampling times.

All coral samples were clustered, and the top 10 OTUs (>1% relative abundance among all coral samples) were selected to construct a heatmap to show the distribution of dominant OTUs among samples (Figure 4). The control samples (Da0, Db0, and Dc0) and samples under the normal light cycle were marked as light treatment (blank circles in Figure 4), and the other samples under dark treatment were labeled as filled circles.

All dark-treated samples were clustered in the rightmost group (Figure 4) except for seven, which were treated under darkness for seven weeks or less. The dark-treated samples in the group had similar bacterial communities, with OTU6 and OTU11 being abundant. These two OTUs were also abundant in five of the seven dark-treated samples outside the rightmost group.

Notably, the heatmap revealed that the relative abundance of OTU6 (*Rhodospirillaceae*) and OTU11 (*Rhizobiales*) increased to become dominant under darkness, whereas the relative abundances of OTU5 (*Alteromonadaceae*) and OTU12 (*Sphingomonas*) were low in the rightmost group and decreased in most of the samples under the dark treatment, except in the Da5 and Dc7 samples.

## Discussion

### Stability of *Endozoicomonas* Abundance During Coral Bleaching

This is the first report to detect quantitative changes in *Endozoicomonas* and total bacterial abundance in bleaching coral cells under a dark treatment using qPCR. This study demonstrated that the abundance of total bacteria and *Endozoicomonas* is stable and high in living bleached corals for 3 months. The *Endozoicomonas-*specific primer from Shiu et al. (2018), modified for use in qPCR, successfully quantified the *Endozoicomonas* variation in coral samples as results were similar to the MiSeq sequencing (bacteria-specific primer). Although Pogoreutz et al. (2017, 2018) reported that the relative *Endozoicomonas* abundance was stable in bleached corals, the authors note that the high relative abundance of *Endozoicomonas* could have been the result of a simultaneous decrease in the number of bacteria and *Endozoicomonas*. Our qPCR results show that *Endozoicomonas* abundance is indeed stable in bleached corals, and the concerns of Pogoreutz and colleagues are not founded.

### Temperature Is Critical for Changes in *Endozoicomonas*

Our results show that, when corals dissociate with Symbiodiniaceae (bleaching), *Endozoicomonas* abundance is not affected under dark treatment. Many studies have shown that Symbiodiniaceae and *Endozoicomonas* abundances decrease during coral bleaching, but these corals were all under heat stress (Bourne et al., 2008; Bayer et al., 2013; Neave et al., 2017; Supplementary Figures S6, S7). Symbiodiniaceae and *Endozoicomonas* were found to be independent on two previous occasions. Shiu et al. (2017) reported high densities of Symbiodiniaceae but a low abundance of *Endozoicomonas* under cold stress, and Pogoreutz et al. (2018) showed low densities of Symbiodiniaceae but a high relative *Endozoicomonas* abundance under excessive nutrient treatment. A decrease in *Endozoicomonas* is therefore suggested to occur when corals are under temperature stress, either hot or cold, regardless of whether the coral is bleached. It is therefore suggested that the majority of *Endozoicomonas* strains are sensitive to temperature stress outside this optimal range.

### *Endozoicomonas* Populations Were Unstable During the Dark Treatment

It is interesting that the most dominant *Endozoicomonas* OTUs shifted more frequently in dark-treated samples than light-treated ones (Supplementary Figure S3A). Although no clear pattern or constant intervals were observed, we propose two reasons for the switches in the most dominant *Endozoicomonas* OTUs: the normal light condition (1) inhibited growth in the OTU9 population or (2) was more beneficial for growing OTU4, and thus OTU4 had a higher relative abundances than OTU9 in the samples.

OTU4 was phylogenetically close to *Endozoicomonas montiporae* strain CL-33, which was isolated from the coral *Montiporae aequituberculata* (Yang et al., 2010), and OTU9 had the best hit in NCBI to *Endozoicomonas euniceicola*, which was isolated from the coral gorgonian *Eunicea fusca* (Pike et al., 2013). *M. aequituberculata* is usually distributed in shallow reef areas with higher light intensity, whereas gorgonians can be distributed in deeper water. This might suggest that OTU4 acclimates better than OTU9 under stronger light conditions, whereas OTU9 acclimates better in dim light conditions than does OTU4.

It is also interesting that OTU470 was a rare population in the present study, and phylogenetically closed to *Endozoicomonas acroporae* strain Acr-14 (Tandon et al., 2018). This strain was isolated from *Acropora* specimens collected at the same time and from the same location, the inlet of the third nuclear power plant, as *E. glabrescens* in this study. Therefore, we suggest that this *Endozoicomonas* OTU470 is *Acropora*-specific (Pollock et al., 2018).

### Identification of Important Environmental Factors

The change in bacterial communities during coral bleaching in oligotrophic reefs may be the result of multiple factors—e.g., light, a decrease in Symbiodiniaceae, and abnormal temperature. However, it is difficult to manipulate only one factor in coral bleaching. By comparing the effects that different factors have on bacterial communities in bleached coral, we can identify the specific environmental factors that change the coral core microbiota and clarify important bacterial candidates for coral health and coral pathogens.

Although corals may not be exposed to long stretches of darkness in natural shallow reef areas, dark experimental manipulation provides a valuable, robust strategy for testing the effects of individual factors on changes in bacterial communities associated with coral bleaching; it also clarifies important bacterial candidates for coral health and identifies the environmental factors critical to the coral core microbiota.

Therefore, although there is a lack of experimental evidence linking specific factors to the reduction in *Endozoicomonas* during coral bleaching, previous studies from us and others (Kurahashi and Yokota, 2007; Bourne et al., 2008; Yang et al., 2010; Pike et al., 2013; Hyun et al., 2014; Shiu et al., 2017; Pogoreutz et al., 2018) suggest that temperature stress or temperature-associated physiological responses of coral may be reason for the reduction in *Endozoicomonas* during coral bleaching.

## Data Availability Statement

The datasets generated for this study can be found in the NCBI SRA PRJNA545213.

## Author Contributions

J-HS contributed to the study design, sampling, molecular experiments, bioinformatics analysis, and manuscript writing and revision. S-PY contributed to the molecular experiments. C-LF and C-JT contributed to the sampling. J-YD contributed to the study design and manuscript revision. T-YF contributed to the study design and the sampling. C-YL contributed to maintain the seawater tanks in the heat experiment. S-LT contributed to the study design, manuscript writing and revision. All authors read and approved the manuscript.

## Conflict of Interest

The authors declare that the research was conducted in the absence of any commercial or financial relationships that could be construed as a potential conflict of interest.

## References

[B1] Bayer T. Neave M. J. Alsheikh-Hussain A. Aranda M. Yum L. K. Mincer T. (2013). The microbiome of the Red Sea coral *Stylophora pistillata* is dominated by tissue-associated *Endozoicomonas bacteria*. *Appl. Environ. Microbiol.* 79 4759–4762. 10.1128/AEM.00695-13 23709513 PMC3719505

[B2] Bourne D. Iida Y. Uthicke S. Smith-Keune C. (2008). Changes in coral-associated microbial communities during a bleaching event. *ISME J.* 2 350–363. 10.1038/ismej.2007.112 18059490

[B3] Bourne D. G. Morrow K. M. Webster N. S. (2016). Insights into the coral microbiome: underpinning the health and resilience of reef ecosystems. *Annu. Rev. Microbiol.* 70 317–340. 10.1146/annurev-micro-102215-095440 27482741

[B4] Bourne D. G. Webster N. S. (2013). “Coral reef bacterial communities,” in *The Prokaryotes: Prokaryotic Communities and Ecophysiology*, eds Rosenberg E. DeLong E. F. Lory S. Stackebrandt E. Thompson F. (Berlin: Springer), 163–187. 10.1007/978-3-642-30123-0_48

[B5] Chen C. P. Tseng C. H. Chen C. A. Tang S. L. (2011). The dynamics of microbial partnerships in the coral *Isopora palifera*. *ISME J.* 5 728–740. 10.1038/ismej.2010.151 20962876 PMC3105734

[B6] Chen H. K. Song S. N. Wang L. H. Mayfield A. B. Chen Y. J. Chen W. N. (2015). A compartmental comparison of major lipid species in a coral-symbiodinium endosymbiosis: evidence that the coral host regulates lipogenesis of its cytosolic lipid bodies. *PLoS One* 10:e0132519. 10.1371/journal.pone.0132519 26218797 PMC4517871

[B7] Chen H. K. Wang L. H. Chen W. U. Mayfield A. B. Levy O. Lin C. S. (2017). Coral lipid bodies as the relay center interconnecting diel-dependent lipidomic changes in different cellular compartments. *Sci. Rep.* 7:3244. 10.1038/s41598-017-02722-z 28607345 PMC5468245

[B8] Denis V. Leung J. K. L. Hsu C. M. Hsieh H. J. Tsai W. S. Chen C. A. (2012). Dark survival of *Oulastrea crispata*. *Galaxea* 14 117–118. 10.3755/galaxea.14.117

[B9] Ding J. Y. Shiu J. H. Chen W. M. Chiang Y. R. Tang S. L. (2016). Genomic insight into the host-endosymbiont relationship of *Endozoicomonas montiporae* CL-33^T^ with its coral host. *Front. Microbiol.* 7:251. 10.3389/fmicb.2016.00251 27014194 PMC4781883

[B10] Edgar R. C. (2013). UPARSE: highly accurate OTU sequences from microbial amplicon reads. *Nat. Methods* 10 996–998. 10.1038/nmeth.2604 23955772

[B11] Glasl B. Herndl G. J. Frade P. R. (2016). The microbiome of coral surface mucus has a key role in mediating holobiont health and survival upon disturbance. *ISME J.* 10 2280–2292. 10.1038/ismej.2016.9 26953605 PMC4989324

[B12] Hoegh-Guldberg O. Smith G. J. (1989). The effect of sudden changes in temperature light and salinity on the population density and export of zooxanthellae from the reef corals *Stylophora pistillata* Esper and *Seriatopora hystrix* Dana. *J. Exp. Ma.r Bioi. Ecol.* 129 279–303. 10.1016/0022-0981(89)90109-3

[B13] Hong M. J. Yu Y. T. Chen C. A. Chiang P. W. Tang S. L. (2009). Influence of species specificity and other factors on bacteria associated with the coral *Stylophora pistillata* in Taiwan. *Appl. Environ. Microbiol.* 75 7797–7806. 10.1128/AEM.01418-09 19854921 PMC2794093

[B14] Hyun D. W. Shin N. R. Kim M. S. Oh S. J. Kim P. S. Whon T. W. (2014). *Endozoicomonas atrinae* sp. nov., isolated from the intestine of a comb pen shell *Atrina pectinata*. *Int. J. Syst. Evol. Microbiol.* 64 2312–2318. 10.1099/ijs.0.060780-0 24733175

[B15] Jorgensen S. L. Hannisdal B. Lanzen A. Baumberger T. Flesland K. Fonseca R. (2012). Correlating microbial community profiles with geochemical data in highly stratified sediments from the Arctic Mid-Ocean Ridge. *Proc. Natl. Acad. Sci. U.S.A.* 109, E2846–2855. 10.1073/pnas.1207574109 23027979 PMC3479504

[B16] Kolde R. (2015). *Pheatmap: Pretty Heatmaps. R Package Version 1.0.2.* Available online at: https://CRAN.R-project.org/package=pheatmap

[B17] Kumar S. Stecher G. Tamura K. (2016). MEGA7: molecular evolutionary genetics analysis version 7.0 for bigger datasets. *Mol. Biol. Evol.* 33 1870–1874. 10.1093/molbev/msw054 27004904 PMC8210823

[B18] Kurahashi M. Yokota A. (2007). *Endozoicomonas elysicola* gen. nov., sp nov., a gamma-proteobacterium isolated from the sea slug *Elysia ornata*. *Syst. Appl. Microbiol.* 30 202–206. 10.1016/j.syapm.2006.07.003 16904280

[B19] Lee S. T. Davy S. K. Tang S. L. Fan T. Y. Kench P. S. (2015). Successive shifts in the microbial community of the surface mucus layer and tissues of the coral *Acropora muricata* under thermal stress. *FEMS Microbiol. Ecol.* 91:fiv142. 10.1093/femsec/fiv142 26564958

[B20] Neave M. J. Apprill A. Ferrier-Pages C. Voolstra C. R. (2016). Diversity and function of prevalent symbiotic marine bacteria in the genus *Endozoicomonas*. *Appl. Microbiol. Biotechnol.* 100 8315–8324. 10.1007/s00253-016-7777-0 27557714 PMC5018254

[B21] Neave M. J. Michell C. T. Apprill A. Voolstra C. R. (2014). Whole-genome sequences of three symbiotic *Endozoicomonas* strains. *Genome Announc.* 2. 10.1128/genomeA.00802-14 25125646 PMC4132622

[B22] Neave M. J. Rachmawati R. Xun L. Michell C. T. Bourne D. G. Apprill A. (2017). Differential specificity between closely related corals and abundant *Endozoicomonas* endosymbionts across global scales. *ISME J.* 11 186–200. 10.1038/ismej.2016.95 27392086 PMC5335547

[B23] Nubel U. Engelen B. Felske A. Snaidr J. Wieshuber A. Amann R. I. (1996). Sequence heterogeneities of genes encoding 16S rRNAs in *Paenibacillus polymyxa* detected by temperature gradient gel electrophoresis. *J. Bacteriol.* 178, 5636–5643. 10.1128/jb.178.19.5636-5643.1996 8824607 PMC178401

[B24] Pantos O. Bongaerts P. Dennis P. G. Tyson G. W. Hoegh-Guldberg O. (2015). Habitat-specific environmental conditions primarily control the microbiomes of the coral *Seriatopora hystrix*. *ISME J.* 9 1916–1927. 10.1038/ismej.2015.3 25668159 PMC4542040

[B25] Pike R. E. Haltli B. Kerr R. G. (2013). Description of *Endozoicomonas euniceicola* sp nov and *Endozoicomonas gorgoniicola* sp nov., bacteria isolated from the octocorals *Eunicea fusca* and *Plexaura* sp., and an emended description of the genus *Endozoicomonas*. *Int. J. Syst. Evol. Microbiol.* 63 4294–4302. 10.1099/ijs.0.051490-0 23832969

[B26] Pogoreutz C. Radecker N. Cardenas A. Gardes A. Voolstra C. R. Wild C. (2017). Sugar enrichment provides evidence for a role of nitrogen fixation in coral bleaching. *Glob. Chang. Biol.* 23 3838–3848. 10.1111/gcb.13695 28429531

[B27] Pogoreutz C. Rädecker N. Cardenas A. Gardes A. Wild C. Voolstra C. R. (2018). Dominance of *Endozoicomonas* bacteria throughout coral bleaching and mortality suggests structural inflexibility of the *Pocillopora verrucosa* microbiome. *Ecol. Evol.* 8 2240–2252. 10.1002/ece3.3830 29468040 PMC5817147

[B28] Pollock F. J. McMinds R. Smith S. David G. B. Bette L. W. Mónica M. (2018). Coral-associated bacteria demonstrate phylosymbiosis and cophylogeny. *Nat. Commun.* 9:4921. 10.1038/s41467-018-07275-x 30467310 PMC6250698

[B29] Schloss P. D. Westcott S. L. Ryabin T. Hall J. R. Hartmann M. Hollister E. B. (2009). Introducing mothur: open-source, platform-independent, community-supported software for describing and comparing microbial communities. *Appl. Environ. Microbiol.* 75 7537–7541. 10.1128/AEM.01541-09 19801464 PMC2786419

[B30] Shikina S. Chiu Y. L. Chung Y. J. Chen C. J. Lee Y. H. Chang C. F. (2016). Oocytes express an endogenous red fluorescent protein in a stony coral, *Euphyllia ancora*: a potential involvement in coral oogenesis. *Sci. Rep.* 6:25868. 10.1038/srep25868 27167722 PMC4863156

[B31] Shiu J. H. Ding J. Y. Tseng C. H. Lou S. P. Mezaki T. Wu Y. T. (2018). A newly designed primer revealed high phylogenetic diversity of *Endozoicomonas* in coral reefs. *Microbes Environ.* 33 172–185. 10.1264/jsme2.ME18054 29760298 PMC6031392

[B32] Shiu J. H. Keshavmurthy S. Chiang P. W. Chen H. J. Lou S. P. Tseng C. H. (2017). Dynamics of coral-associated bacterial communities acclimated to temperature stress based on recent thermal history. *Sci. Rep.* 7:14933. 10.1038/s41598-017-14927-3 29097716 PMC5668310

[B33] Tandon K. Chiang P. W. Chen W. M. Tang S. L. (2018). Draft genome sequence of *Endozoicomonas acroporae* strain Acr-14T, isolated from Acropora coral. *Genome Announc.* 6:e01576-17. 10.1128/genomeA.01576-17 29439049 PMC5805887

[B34] Tandon K. Lu C. Y. Chiang P. W. Wada N. Yang S. H. Chan Y. F. (2020). Comparative genomics: dominant coral-bacterium *Endozoicomonas acroporae* metabolizes dimethylsulfoniopropionate (DMSP). *Isme J.* 14, 1290–1303. 10.1038/s41396-020-0610-x 32055028 PMC7174347

[B35] Wang Q. Garrity G. M. Tiedje J. M. Cole J. R. (2007). Naive Bayesian classifier for rapid assignment of rRNA sequences into the new bacterial taxonomy. *Appl. Environ. Microbiol.* 73 5261–5267. 10.1128/AEM.00062-07 17586664 PMC1950982

[B36] William S. Clayton J. Lasker H. R. (1982). Effects of light and dark treatments on feeding by the reef coral *Pocillopora damicornis*. *J. Exp. Mar. Biol. Ecol.* 63 269–279. 10.1016/0022-0981(82)90183-6

[B37] Wilson K. (2001). Preparation of genomic DNA from bacteria. *Curr. Protoc. Mol. Biol.* 56 2.4.1–2.4.5. 10.1002/0471142727.mb0204s56 18265184

[B38] Yang C. S. Chen M. H. Arun A. B. Chen C. A. Wang J. T. Chen W. M. (2010). *Endozoicomonas montiporae* sp. nov., isolated from the encrusting pore coral *Montipora aequituberculata*. *Int. J. Syst. Evol. Microbiol.* 60 1158–1162. 10.1099/ijs.0.014357-0 19666790

[B39] Yonge C. M. Nicholls A. G. (1931). Studies on the physiology of corals. V. The effect of starvation in light and darkness on the relationship between corals and zooxanthellae. *Sci. Rep.* 1 177–211.

[B40] Ziegler M. Seneca F. O. Yum L. K. Palumbi S. R. Voolstra C. R. (2017). Bacterial community dynamics are linked to patterns of coral heat tolerance. *Nat. Commun.* 8:14213. 10.1038/ncomms14213 28186132 PMC5309854

