# Supplementary material for: Shifting in the Dominant Bacterial Group Endozoicomonas Is Independent of the Dissociation With Coral Symbiont Algae
Source: Front Microbiol. 2020 Jul 31;11:1791. doi: 10.3389/fmicb.2020.01791 (PMC7412130; doi:10.3389/fmicb.2020.01791)
Supplement: Supplementary file 1 [file Table_1.DOCX]

**Supplementary Document**

**Shifting in the Dominant Bacterial Group *Endozoicomonas* Is Independent to the Dissociation with Coral Symbiont Algae**

Jia-Ho Shiu^123^, Sheng-Ping Yu^2^, Chia-Ling Fong^2^, Jiun-Yan Ding^2^, Chih-Jui Tan^4^, Tung-Yung Fan^4^, Chih-Ying Lu^2^, Sen-Lin Tang^12^

*^1^Molecular and Biological Agricultural Sciences Program, Taiwan International Graduate Program, Academia Sinica, Taipei, Taiwan, and National Chung-Hsing University, Taichung, Taiwan; ^2^Biodiversity Research Center, Academia Sinica, Taipei, Taiwan; ^3^Graduate Institute of Biotechnology, National Chung-Hsing University, Taichung, Taiwan; ^4^National Museum of Marine Biology and Aquarium, Pingtung, Taiwan*

Correspondence: Sen-Lin Tang

Telephone: +886-2-27893863

Facsimile: +886-2-27890844

E-mail: sltang@gate.sinica.edu.tw

Address: Biodiversity Research Center, Academia Sinica, No. 128 Sec. 2, Academia Rd., Nankang, Taipei 11529, Taiwan

Running title: *Endozoicomonas* unchanged in coral bleaching

Key words: coral microbe, coral bleaching, *Endozoicomonas*, Symbiodiniaceae

**Materials and Methods**

**Husbandry system**

Two fragments from the same colony were placed in two 20-liter tanks using the same seawater circulation system. After acclimating (1 week), one of the fragments was treated under a light/dark cycle (8 h light/16 h dark) and the other was covered for a dark treatment (24 h dark) for 15 weeks (Figure 1A). There are total of six fragments from three colonies in six tanks and three independent seawater circulation systems. To maintain oligotrophic conditions, we did not feed *Euphyllia*. We used unfiltered seawater from the northeast coast of Taiwan in the system. Two liters of seawater was changed from each tank every 2–3 days.

**Heat experiment**

We collected three colonies of *Euphyllia glabrescens* more than 10 m apart at a depth of 3.5 m from the power plant inlet in Kenting, Taiwan (21°57'21.6"N 120°45'18.1"E). Colonies were transported to a laboratory in Academia Sinica, Taipei. Each colony was separated into two fragments, which were divided into two tanks with independent seawater circulation systems. After acclimating (1 week), one of the fragments was kept at 25°C as the control and the other was treated with an increased temperature (+1°C /day) until 31°C. Coral tentacles were sampled before and after three days of 31°C treatment. At each sampling time, three coral tentacles from each fragment were cut using scissors and stored in 99% ethanol at −80°C for DNA extraction and comparative quantitative PCR (qPCR). The qPCR protocols were the same as those in the main text.

**Figure legend**

Figure S1. *Euphyllia* *glabrescens* bleached under the dark treatment. The photo on the left shows colonies before the dark treatments. The photo on the right shows the three colonies after 15 weeks of dark treatment, and the top left colony was under a normal light cycle treatment.

Figure S2. Fold-changes in *Endozoicomonas* relative abundances in colonies a, b, and c under the dark treatment based on MiSeq (A) and comparative qPCR (B) results. For the comparative qPCR results, the copy numbers of the *Endozoicomonas* 16S rRNA gene were normalized by the copy numbers of total bacterial 16S rRNA genes. For the MiSeq results, the relative abundances were divided by those in samples before the dark treatment (Da0, Db0, and Dc0).

Figure S3. (A) The bubble plot for variation in the relative abundances of three dominant *Endozoicomonas* OTUs: OTU4, OTU9, and OTU120. The bubble size represents the relative abundance of each *Endozoicomonas* OTU in each sample. The x axis lists sampling times and the y axis lists the five coral sub-colonies with high relative *Endozoicomonas* abundances. Solid circles indicate dark-treated samples and blank circles indicate normal light treatment samples. (B) Phylogenetic tree of *Endozoicomonas* 16S rRNA sequences. The phylogeny was constructed with the four representative *Endozoicomonas* OTU sequences of the 16S rRNA gene from this study (black circle) and other representative *Endozoicomonas*, *Spongiobacter,* and outgroup sequences downloaded from GenBank. Numbers on branches are bootstrap values (1000 bootstraps; < 50% not shown). The scale bar corresponds to 0.01 substitutions per nucleotide position. (C) Phylogenetic tree of *Endozoicomonas* full length 16S rRNA sequences using the Maximum Likelihood method and Tamura-Nei model. The phylogeny was constructed with the *Endozoicomonas* type strain sequences of the 16S rRNA gene and outgroup sequences downloaded from GenBank. The percentage of trees in which the associated taxa clustered together is shown next to the branches. The scale bar corresponds to 0.02 substitutions per nucleotide position.

Figure S4. Variation in bacterial communities among coral and seawater samples in hierarchical clustering (CLUSTER) analysis (A) and bacterial taxonomy at the order level (B). (A) Bacterial communities varied between coral and seawater samples based on complete linkage among samples. Symbols indicate bacterial communities collected from coral or seawater samples. CLUSTER was based on a Bray-Curtis similarity matrix constructed from the relative abundance of OTUs in each sample. (B) Bacterial taxonomic composition at the order level in all coral and seawater samples.

Figure S5. Variation in bacterial communities among coral samples under different treatments in hierarchical clustering (CLUSTER) analysis. Different symbols indicate coral samples under different treatments. CLUSTER was based on a Bray-Curtis similarity matrix constructed from the relative abundance of OTUs in each sample.

Figure S6. Photos of the *Euphyllia glabrescens* colonies before and after the heat treatment (31°C).

Figure S7. Comparative qPCR results for fold-change in the abundance of *Symbiodiniaceae* ITS (A), and the *Endozoicomonas* 16S rRNA gene (B) normalized with the *Euphyllia glabrescens* beta-actin gene.

Figure S8. Bacterial composition (genus level; g_) in coral samples. Bacterial taxonomies that could not be assigned to the genus level from the SILVA database are represented as their higher family (f_), order (o_), class (c_), or phylum (p_) taxonomic name.

Figure S1


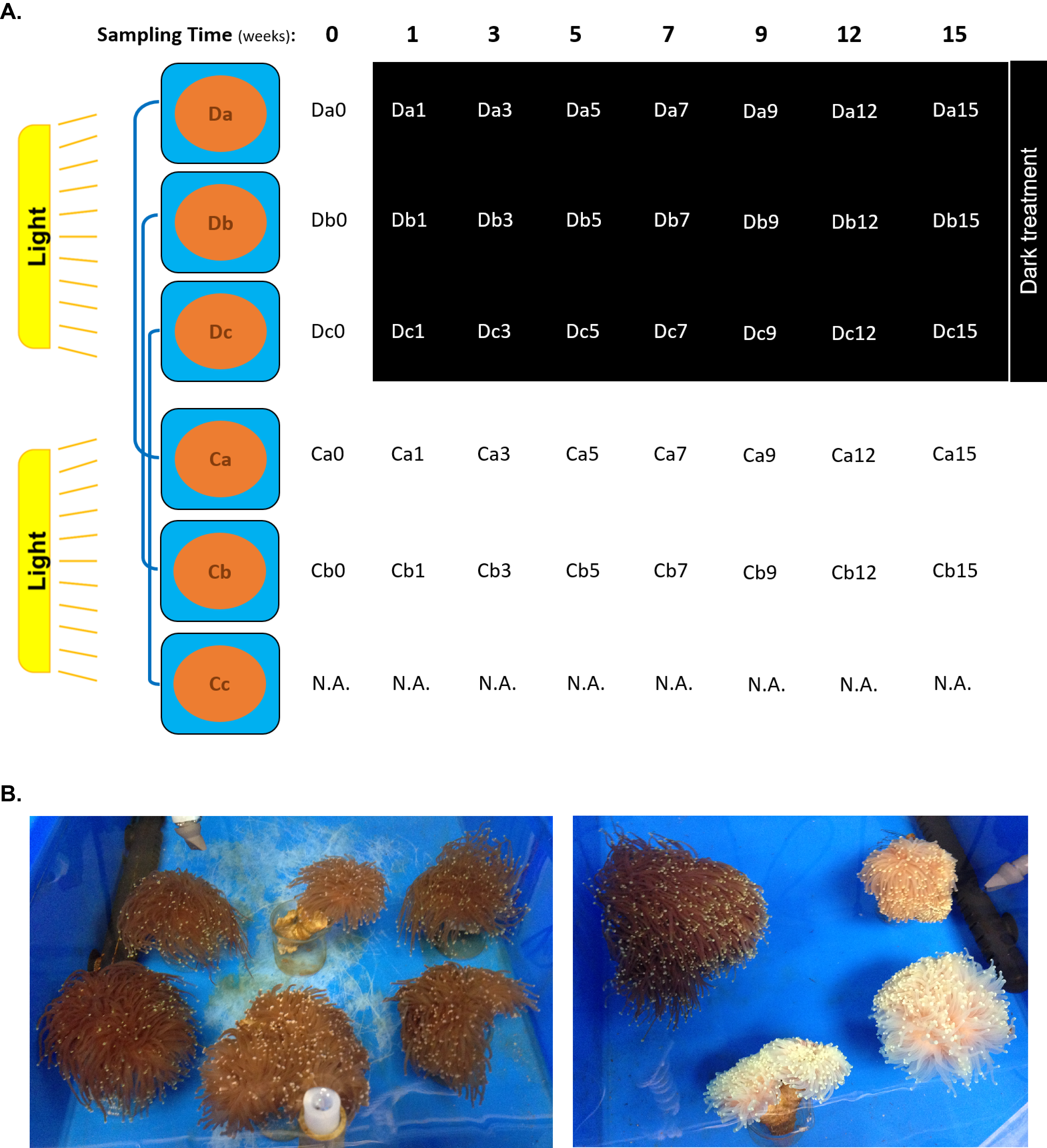


Figure S2.


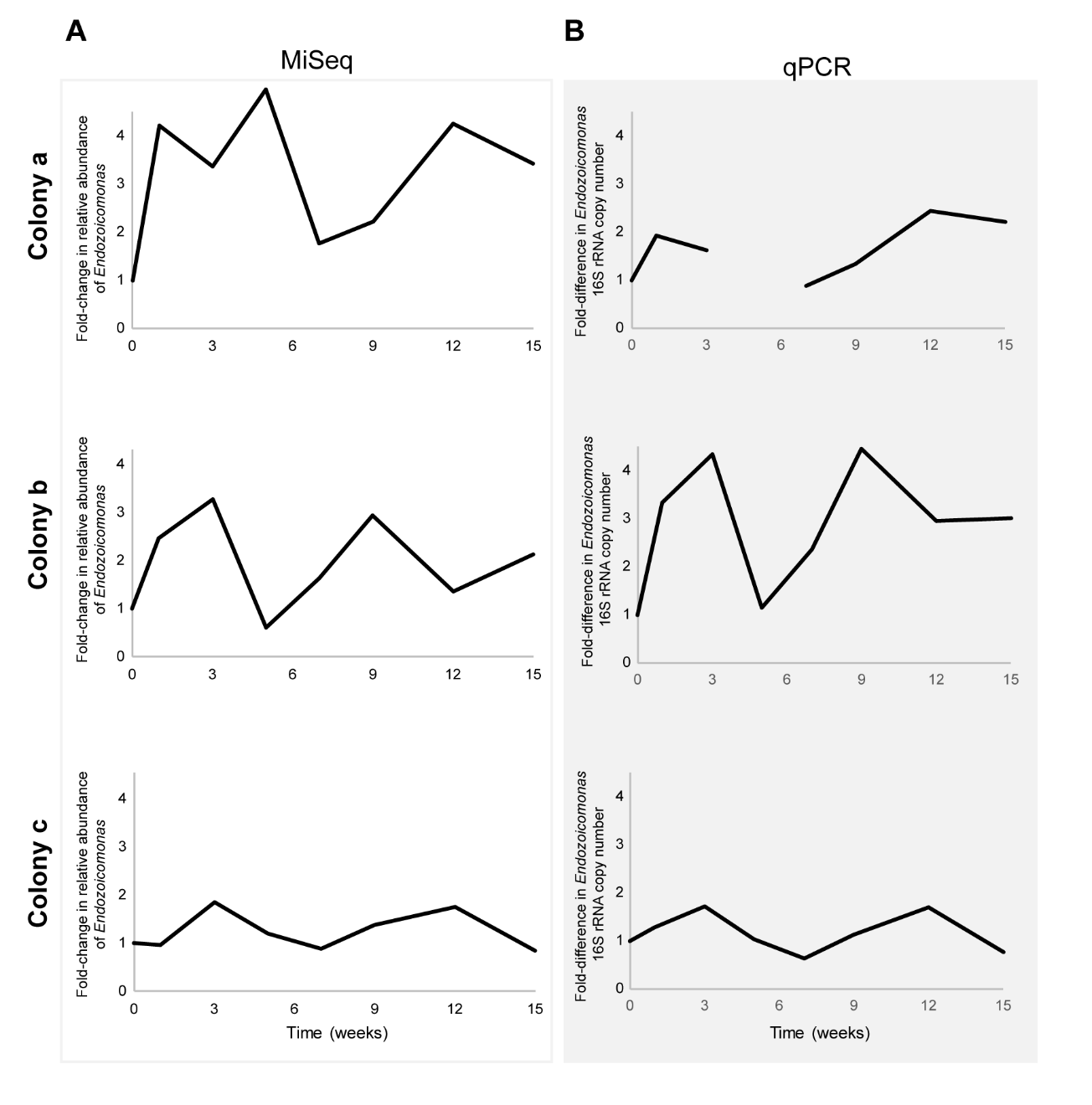


Figure S3


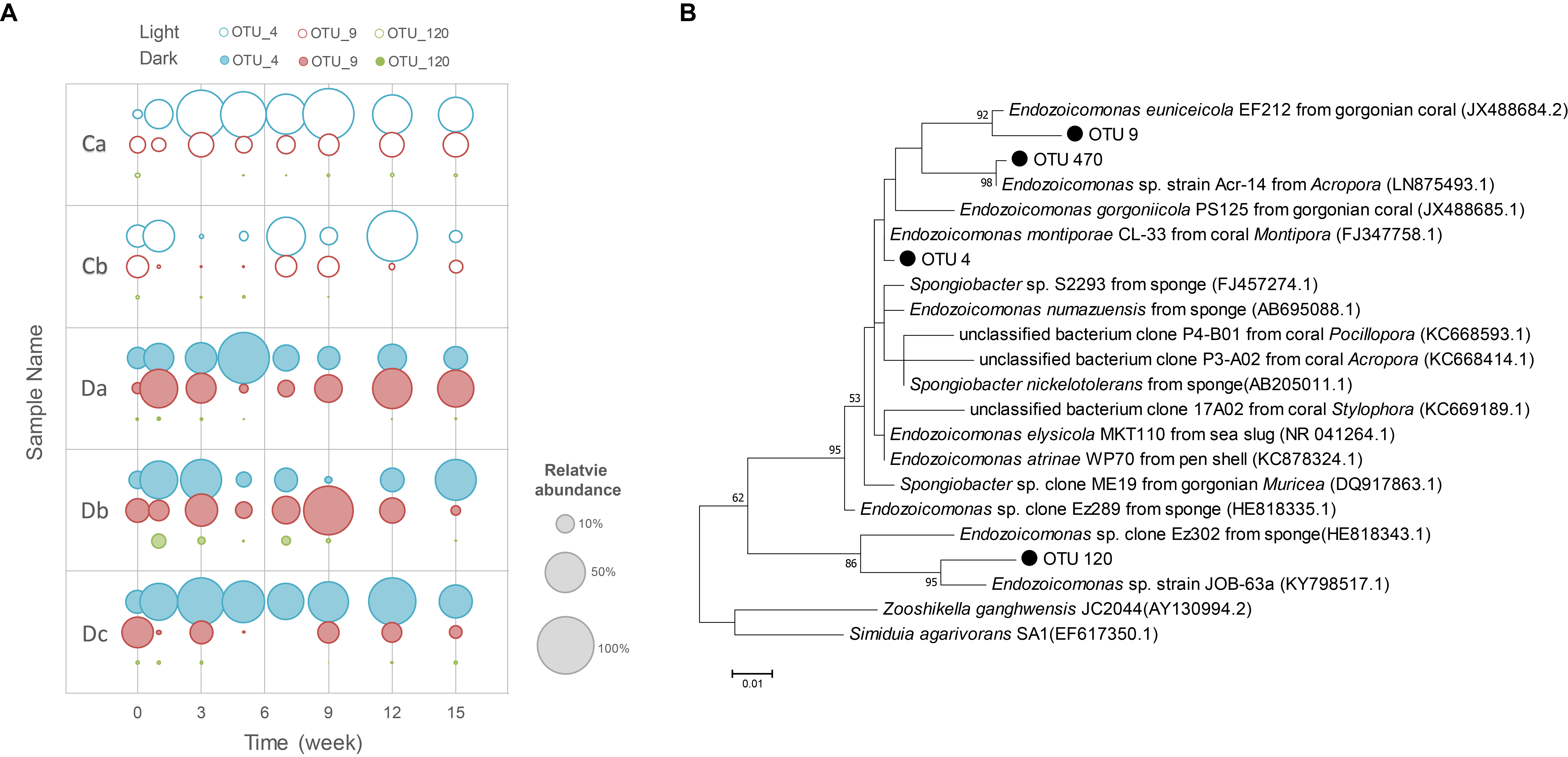


**C**

Figure S4


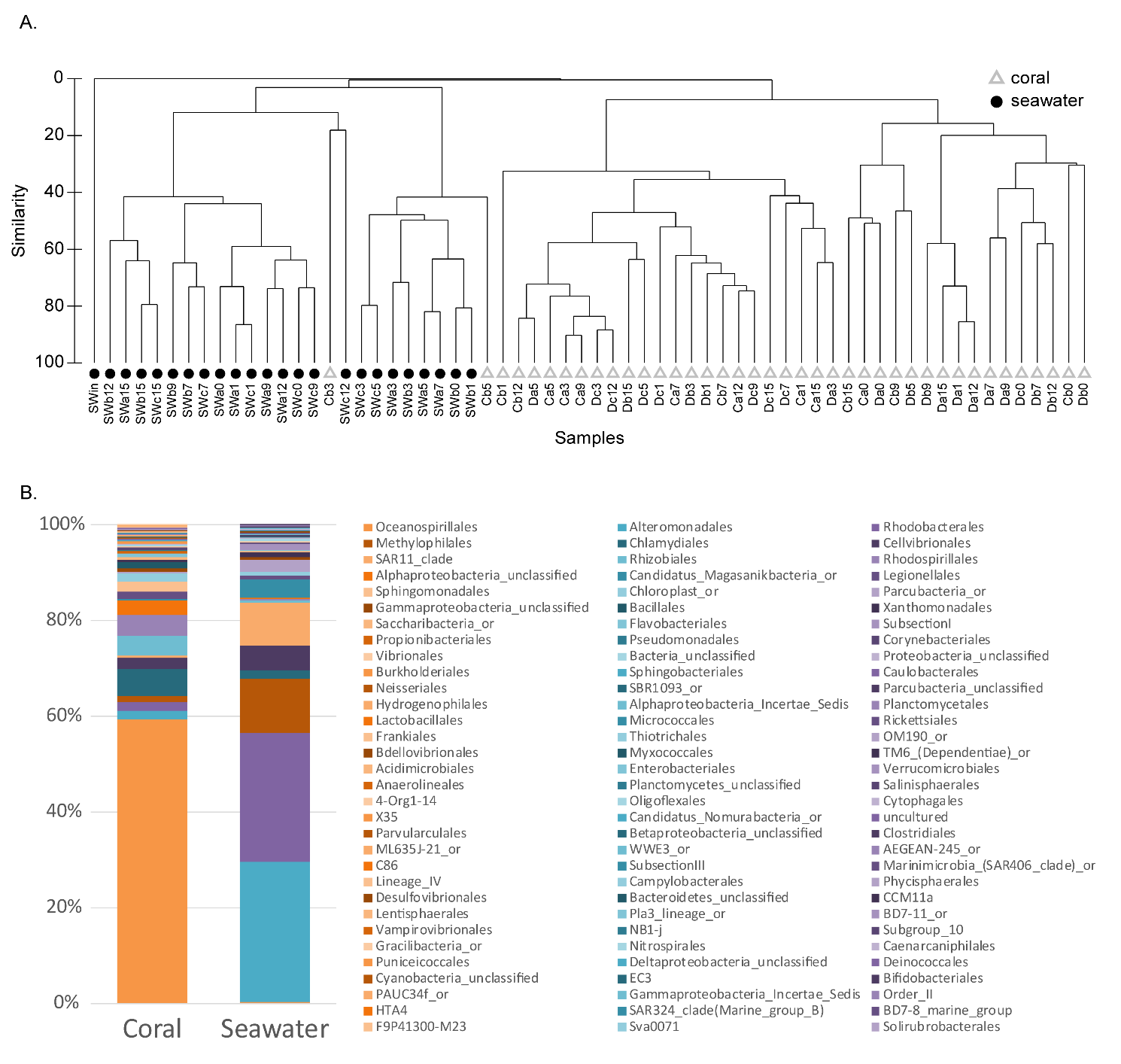


Figure S5.


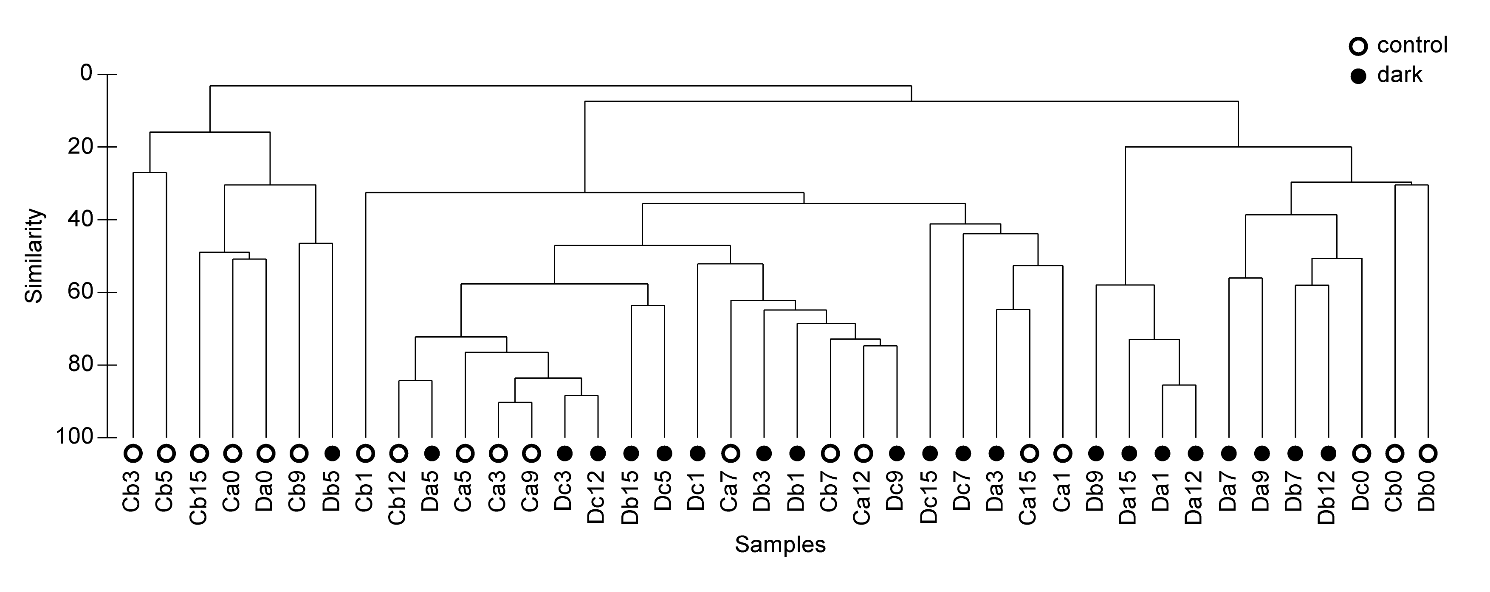


Figure S6.

| Colony | Heat samples_Before | Heat samples_After | Control samples_Before | Control samples_After |
| --- | --- | --- | --- | --- |
| **a** | 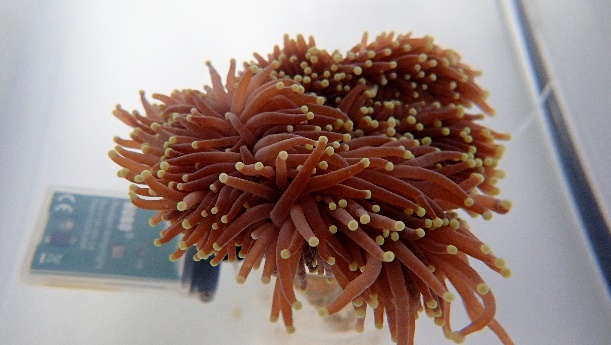 | 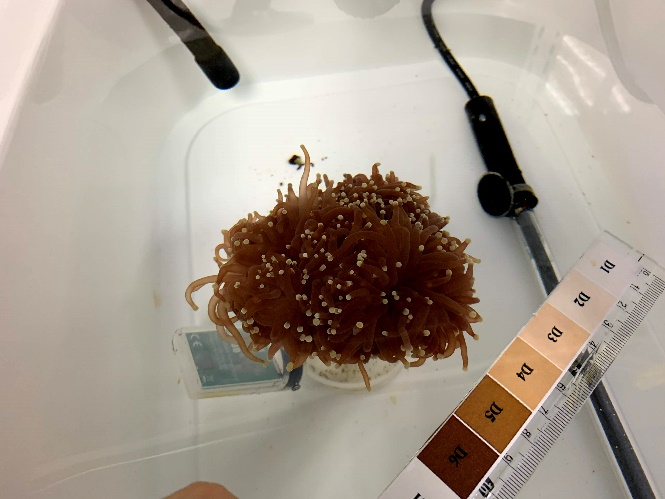 | 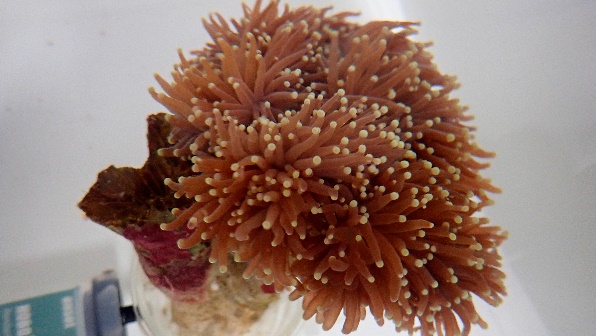 | 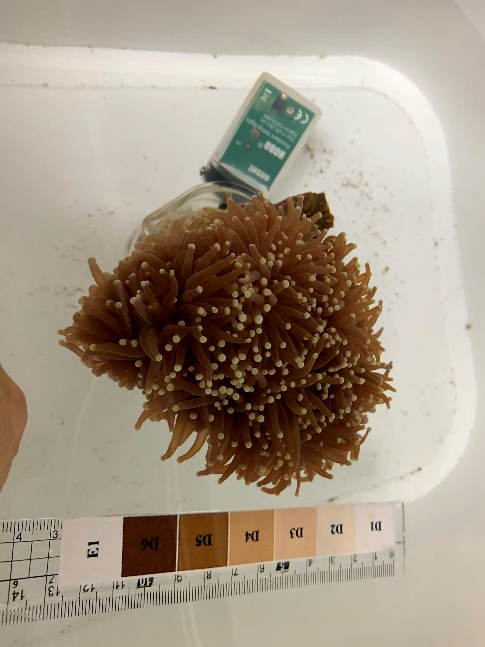 |
| **b** | 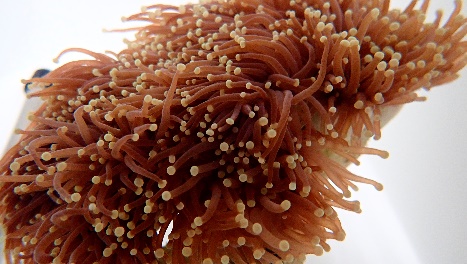 | 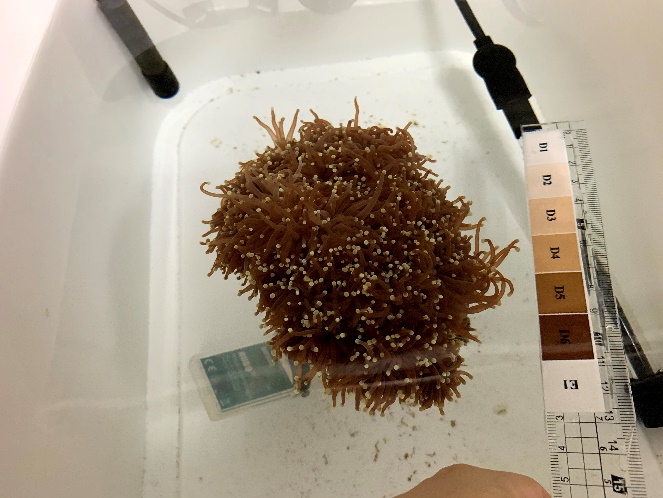 | 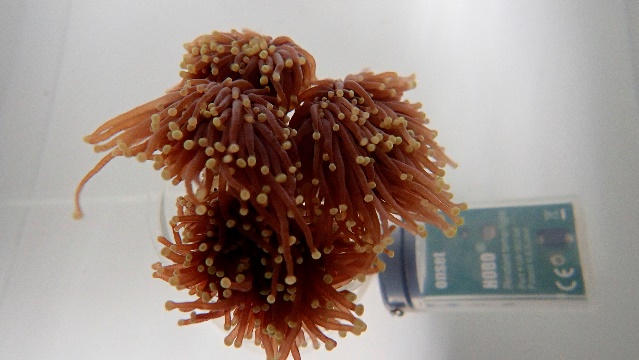 | 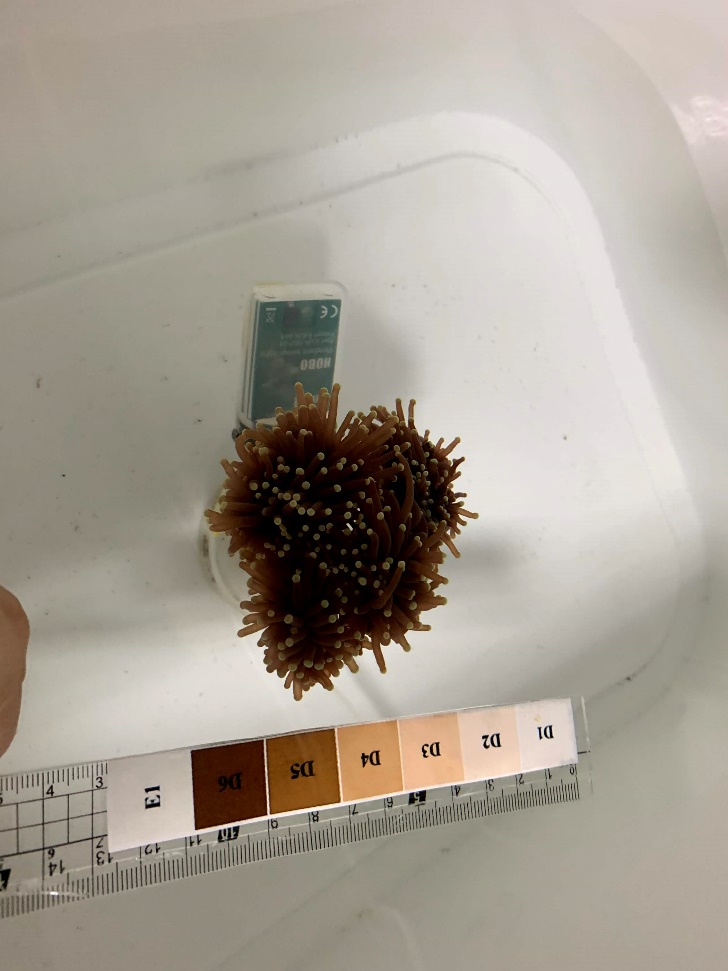 |
| **c** | 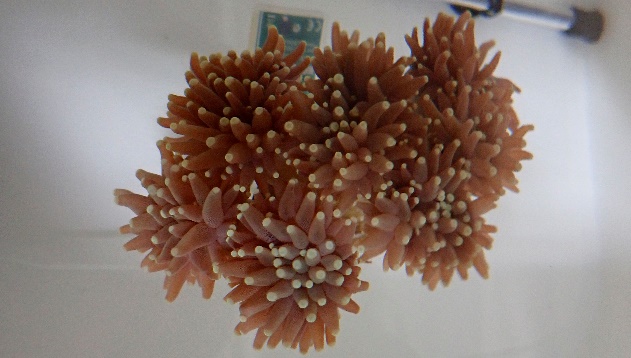 | 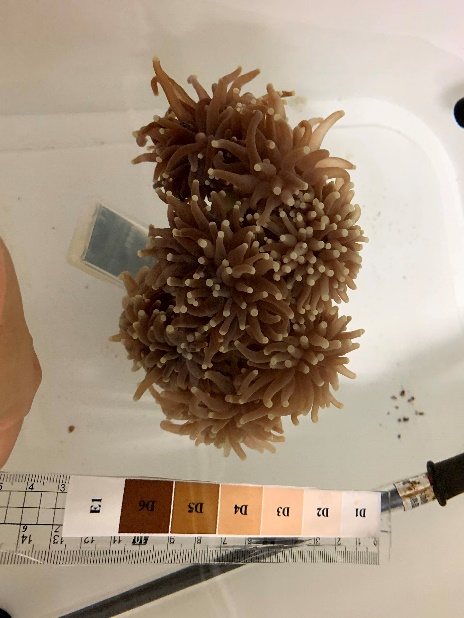 | 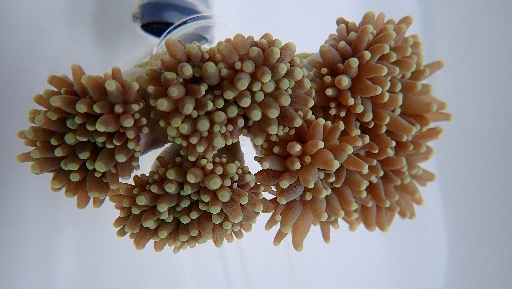 | 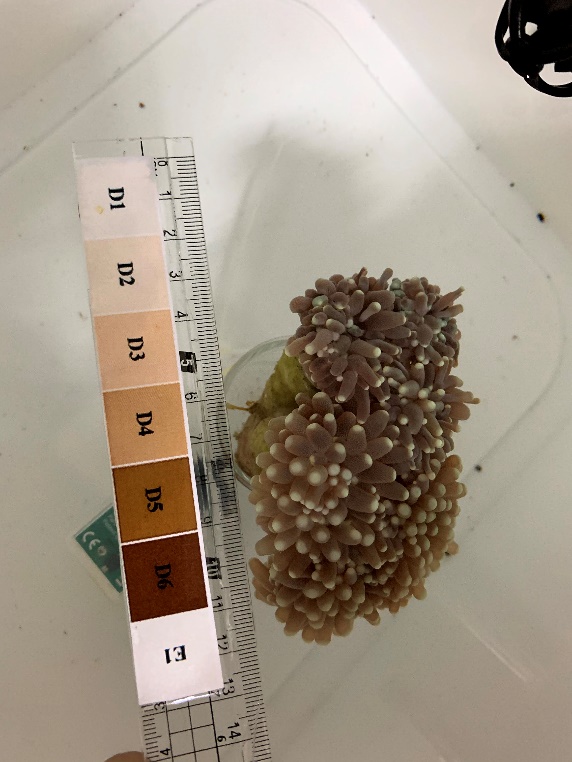 |

Figure S7.


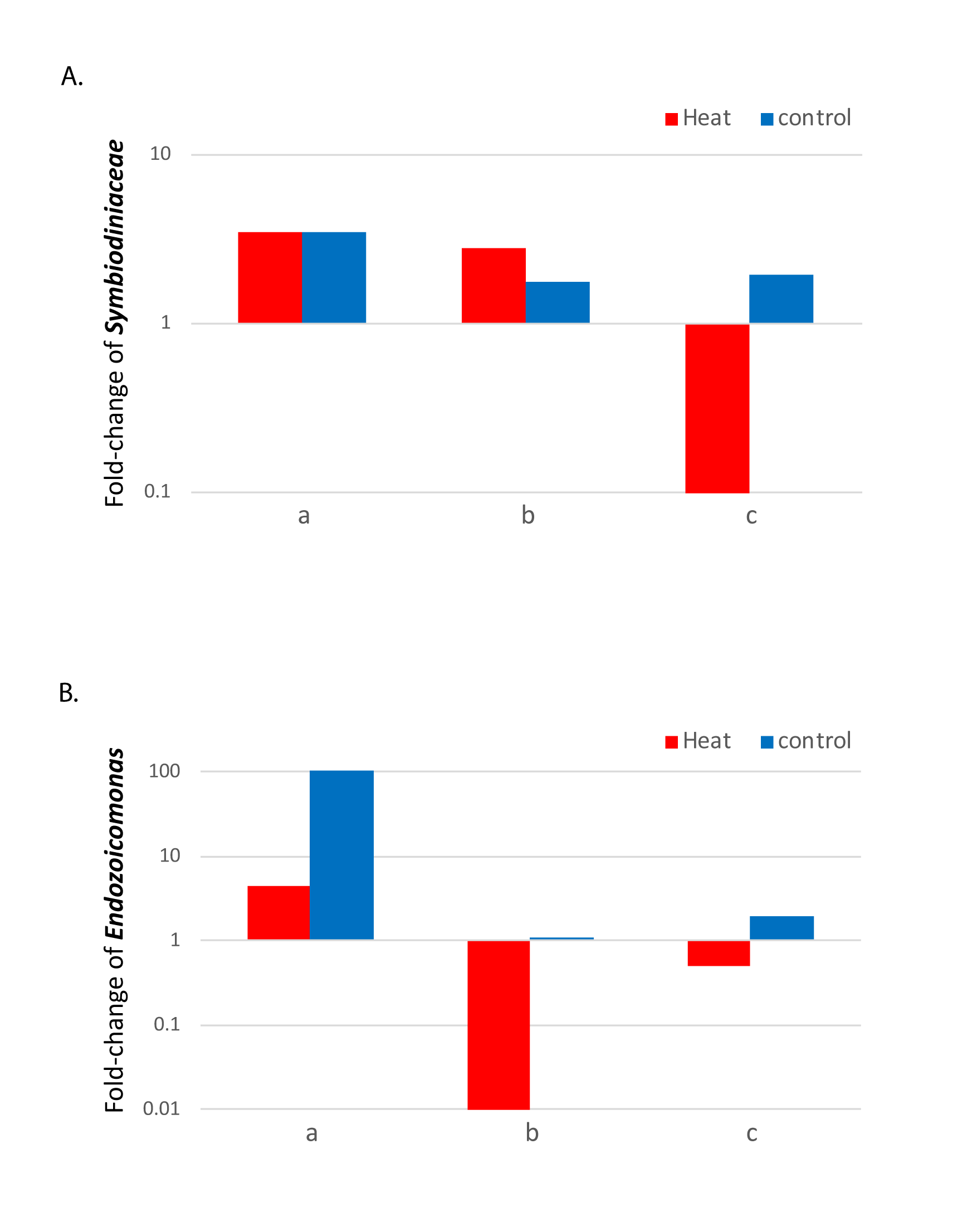


Figure S8.


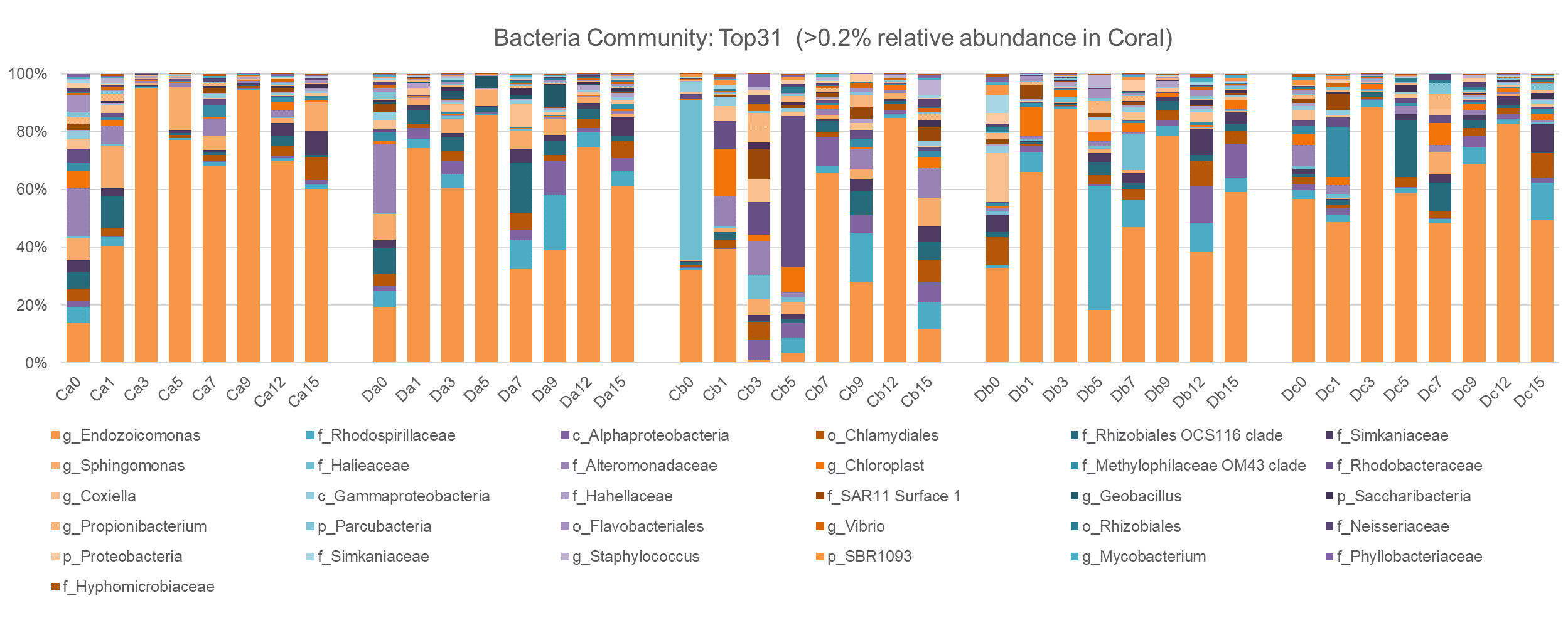


Table S1. Sample names of coral tentacles and seawater collected from the seawater tanks under treatment. For each sample, three tentacles from each *Euphyllia glabrescence* colony or 1 L seawater were collected for MiSeq sequencing and qPCR.

| **Treatment** | **Colony** | **Experiment Time (weeks)** | | | | | | | |
| --- | --- | --- | --- | --- | --- | --- | --- | --- | --- |
|  |  | **0** | **1** | **3** | **5** | **7** | **9** | **12** | **15** |
| **Dark** | a | Da0 | Da1 | Da3 | Da5* | Da7 | Da9 | Da12 | Da15 |
|  | b | Db0 | Db1 | Db3 | Db5 | Db7 | Db9 | Db12 | Db15 |
|  | c | Dc0 | Dc1 | Dc3 | Dc5 | Dc7 | Dc9 | Dc12 | Dc15 |
| **Light** | a | Ca0 | Ca1 | Ca3 | Ca5 | Ca7 | Ca9 | Ca12 | Ca15 |
|  | b | Cb0 | Cb1 | Cb3 | Cb5 | Cb7 | Cb9 | Cb12 | Cb15 |
|  | c | N.A. | N.A. | N.A. | N.A. | N.A. | N.A. | N.A. | N.A. |
| **Seawater** |  | SWa0 | SWa1 | SWa3 | SWa5 | SWa7 | SWa9 | SWa12 | SWa15 |
|  | SWin | SWb0 | SWb1 | SWb3 | N.A. | SWb7 | SWb9 | SWb12 | SWb15 |
|  |  | SWc0 | SWc1 | SWc3 | SWc5 | SWc7 | SWc9 | SWc12 | SWc15 |

Abbreviations in sample names: The first character represents the seawater sample (SW), coral samples under the dark treatment (D), or coral samples exposed to the normal light cycle (L) in the circulation system; the next character represents samples from the circulation system (a, b, or c) and the last number is sampling time – 0: before treatment, 1: 1 week, 3: 3 weeks, 5: 5 weeks, etc. after treatment. N.A.: data missing. *Only MiSeq sequencing data were collected for the bacterial community at this sampling time.

Table S2. Annotation of the top 10 OTUs after excluding *Endozoicomonas* OTUs.

| **OTU** | **Taxonomy in SILVA database** | **Accession number of best BLAST** | **% identity** | **Taxonomy in NCBI** |
| --- | --- | --- | --- | --- |
| **OTU6** | *Alphaproteobacteria;Rhodospirillales;Rhodospirillaceae* | NR_136430 | 90.62 | *Emcibacter nanhaiensis* |
| **OTU11** | *Alphaproteobacteria;Rhizobiales;OCS116_clade* | NR_044447.1 | 94.16 | *Maritalea mobilis* |
| **OTU15** | *Cyanobacteria* | NR_117840.1 | 78.77 | *Fusobacterium nucleatum* |
| **OTU8** | *Gammaproteobacteria;Cellvibrionales;Halieaceae;* | NR_125526.1 | 95.83 | *Luminiphilus syltensis* |
| **OTU7** | *Betaproteobacteria;Methylophilales;Methylophilaceae* | NR_043690.1 | 95.1 | *Methylotenera mobilis* |
| **OTU10** | *Alphaproteobacteria;SAR11_clade;Surface_1* | NR_074224.1 | 97.8 | *Candidatus Pelagibacter ubique* |
| **OTU3** | *Alphaproteobacteria;Rhodobacterales;Rhodobacteraceae* | NR_116682.1 | 97.42 | *Marivita byunsanensis* |
| **OTU19** | *Alphaproteobacteria* | NR_136430.1 | 90.35 | *Emcibacter nanhaiensis* |
| **OTU5** | *Gammaproteobacteria;Alteromonadales;Alteromonadaceae* | NR_156088.1 | 94.83 | *Alteromonas lipolytica* |
| **OTU12** | *Alphaproteobacteria;Sphingomonadales;Sphingomonadaceae;Sphingomonas* | NR_104893.1 | 100 | *Sphingomonas paucimobilis* |
